# Updates in pathobiological aspects of anaplastic large cell lymphoma

**DOI:** 10.3389/fonc.2023.1241532

**Published:** 2023-09-22

**Authors:** Rui Wu, Megan S. Lim

**Affiliations:** Department of Pathology and Laboratory Medicine, Memorial Sloan Kettering Cancer Center, New York, NY, United States

**Keywords:** pathogenesis, ALCL, lymphoma biology, genetic abnormalities, proteomics

## Abstract

Anaplastic large cell lymphomas (ALCL) encompass several distinct subtypes of mature T-cell neoplasms that are unified by the expression of CD30 and anaplastic cytomorphology. Identification of the cytogenetic abnormality t(2;5)(p23;q35) led to the subclassification of ALCLs into ALK+ ALCL and ALK- ALCL. According to the most recent World Health Organization (WHO) Classification of Haematolymphoid Tumours as well as the International Consensus Classification (ICC) of Mature Lymphoid Neoplasms, ALCLs encompass ALK+ ALCL, ALK- ALCL, and breast implant-associated ALCL (BI-ALCL). Approximately 80% of systemic ALCLs harbor rearrangement of *ALK*, with *NPM1* being the most common partner gene, although many other fusion partner genes have been identified to date. ALK- ALCLs represent a heterogeneous group of lymphomas with distinct clinical, immunophenotypic, and genetic features. A subset harbor recurrent rearrangement of genes, including *TYK2*, *DUSP22*, and *TP63*, with a proportion for which genetic aberrations have yet to be characterized. Although primary cutaneous ALCL (pc-ALCL) is currently classified as a subtype of primary cutaneous T-cell lymphoma, due to the large anaplastic and pleomorphic morphology together with CD30 expression in the malignant cells, this review also discusses the pathobiological features of this disease entity. Genomic and proteomic studies have contributed significant knowledge elucidating novel signaling pathways that are implicated in ALCL pathogenesis and represent candidate targets of therapeutic interventions. This review aims to offer perspectives on recent insights regarding the pathobiological and genetic features of ALCL.

## 1 Introduction

**Figure 1:**
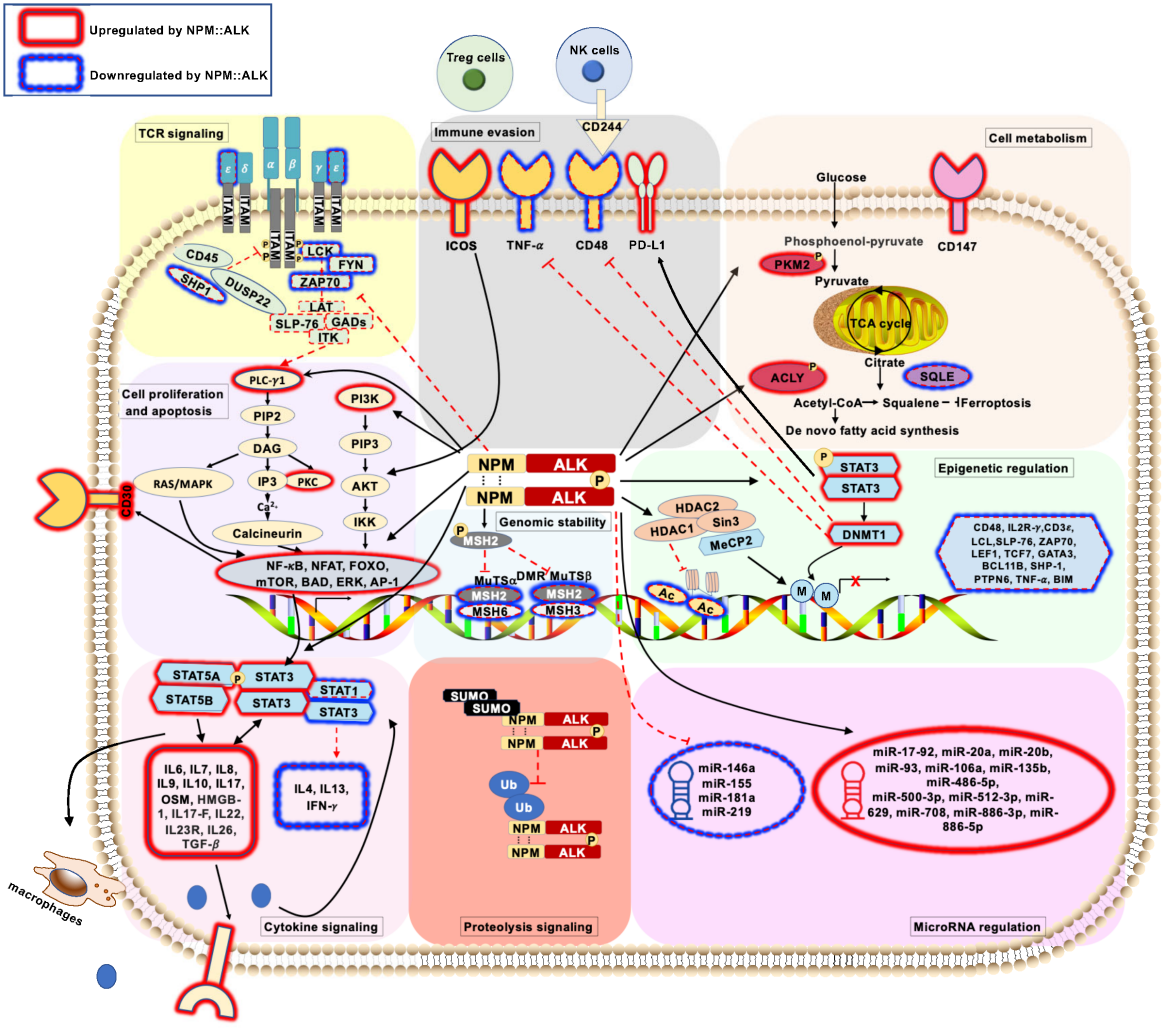
Schematic representation of pathogenic signaling pathways in ALK+ ALCL (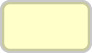) represents deregulated T-cell receptor signaling pathways mediated by *NPM::ALK*. (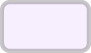) represents deregulated cell proliferation and apoptosis signaling pathways mediated by *NPM::ALK*. (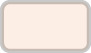) represents deregulated cellular metabolism signaling pathways mediated by *NPM::ALK*. (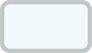) represents deregulated genomic stability signaling pathways mediated by *NPM::ALK*. (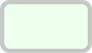) represents deregulated epigenetic regulation signaling pathway mediated by *NPM::ALK*. (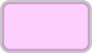) represents deregulated miRNA repertoire by *NPM::ALK*. (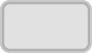) represents immune evasion mechanisms mediated by *NPM::ALK*. (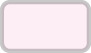) represents deregulated cytokine signaling mediated by *NPM::ALK*. (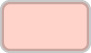) represents deregulated proteolytic signaling mediated by *NPM::ALK*.

Anaplastic large cell lymphomas (ALCL) refer to a heterogeneous group of CD30-positive T-cell neoplasms with diverse clinical, histologic, and genetic features. The disease group comprises approximately 15% of all peripheral T-cell lymphoma and 3 to 5% of all non-Hodgkin lymphoma (1). ALCL was first recognized in 1985 based on the large size of the neoplastic cells with uniform strong expression of CD30 (2). The recurrent chromosomal translocation t (2;5)(p23;q35), which was identified in 1994, results in a novel fusion tyrosine kinase involving the N-terminus of the nucleophosmin (*NPM1*) gene and the C-terminus of the anaplastic lymphoma kinase (*ALK*) gene (3). The chimeric protein NPM::ALK functions as an oncogenic tyrosine kinase which impacts diverse cellular signaling pathways leading to lymphoma. In addition to *NPM1*, over 20 distinct partner genes of *ALK* have been identified (**Figure 1**) (4). Moreover, the significantly enhanced survival of ALK+ ALCL patients and distinct genetic features rationalized its distinction from ALK- ALCLs (5, 6). Based on the recent understanding of the genetic basis of ALK- ALCL and those that occur in breast-implant ALCL, the updated 5^th^ edition of the World Health Organization Classification of Haematolymphoid Tumours (5), and the International Consensus Classification (ICC) of Mature Lymphoid Neoplasms (7) recognize three subtypes, namely ALK+ ALCL, ALK- ALCL, breast implant-associated ALCL (BI-ALCL) (5). Further, we discuss the pathobiological features of primary cutaneous anaplastic large cell lymphoma (pc-ALCL) due to many overlapping histologic and immunophenotypic features with other ALCLs. Recent genomic studies provide an enhanced understanding of the pathobiological events in this group of intriguing neoplasms that will be summarized in this manuscript (6, 8).

## 2 Pathobiology of ALK+ ALCL

### 2.1 *ALK* rearrangements in ALK+ ALCL

ALK+ ALCLs, by definition, express the ALK protein, which functions as a strong driver oncogene. *ALK* gene rearrangements result in the expression of a novel fusion protein and constitutive activation of the ALK tyrosine kinase (9). The majority (80%) of cases express *NPM::ALK* as a result of t(2;5)(q23;35) involving the 3’ segment of the *ALK* gene on chromosome 2p23 and the 5’ segment of the nucleophosmin (*NPM1*) gene on chromosome 5q35. The chimeric protein NPM::ALK is composed of the N-terminal oligomerization domain of NPM1 and the C-terminal tyrosine kinase domain of ALK. Due to ligand-independent oligomerization mediated by NPM1, the catalytic domain of ALK undergoes transautophosphorylation with constitutive tyrosine kinase activity, which translates into increased intracellular signaling promoting cell proliferation, resistance to apoptosis, and oncogenic transformation (10). Apart from *NPM1* as its N-terminal fusion partner, in approximately 20% of ALK+ ALCLs, *ALK* is fused with other genes, including tropomyosin 3 and 4 (*TPM3* and *TPM4*) (11), TRK-fused gene (*TFG*) (12), 5-amino-imidazole-4-carboxamide ribonucleotide formyltransferase/IMP cyclohydrolase (*ATIC*) (13), ring finger protein 213 (*RNF213*) (14), clathrin heavy chain (*CLTC*) (14), moesin (*MSN*) (15), non-muscle myosin heavy chain (*MYH9*) (15), TNF receptor-associated factor 1 (*TRAF1*) (16), eukaryotic translation elongation factor I gamma (*EEF1G*) (17) and the poly(A) binding protein cytoplasmic 1 (*PABPC1*) (18). In contrast to NPM::ALK, which is expressed in both the nucleus and cytoplasm, other ALK fusion proteins are localized in various cellular compartments (**Table 1**) (3, 11, 13–20). *TPM3::ALK*, the second most frequent fusion, is present in approximately 15% of ALK+ ALCLs. Despite all *ALK* rearrangements involving the same region of *ALK*, the downstream signaling pathways vary due to the different fusion partners (21). The pathogenetic mechanisms regulated by variant *ALK* fusion genes have not been explored due to the rarity of cases.

**Table 1:** Summary of chromosomal rearrangements in ALK+ ALCL.

ALK fusion proteins	Translocation	Localization	Reference
NPM::ALK	t(2;5)(p23;q35)	nucleus, cytoplasm	(3)
TPM3::ALK	t(1;2)(q25; p23)	cytoplasm	(11)
TPM4::ALK	t(2;19)(p23;p13)	cytoplasm	(11)
TFG::ALK	t(2;3)(p23;q21)	cytoplasm	(12)
ATIC::ALK	inv(2)(p23q35)	cytoplasm	(13)
RNF213::ALK	t(2;17)(p23;q25)	cytoplasm	(14)
CLTC::ALK	t(2;17)(p23;q23)	cytoplasm	(14)
MSN::ALK	t(X;2)(q11; p23)	cytoplasm	(15)
MYH9::ALK	t(2;22)(p23;q11)	cytoplasm	(15)
TRAF1::ALK	t(2;19)(p23;q33)	cytoplasm	(16)
EEF1G::ALK	t(2;11)(p23;q11)	cytoplasm	(17)
PABPC1::ALK	t(2;9)(p23;q33)	cytoplasm	(18)

### 2.2 Deregulated T-cell receptor signaling pathway in ALK+ ALCL

T-cell receptor (TCR) engagement triggers various cascades of signaling pathways, including phospholipase C-γ1 (PLC-γ1)-inositol triphosphate (IP3)-Ca^2+^-nuclear factor of activated T-cells (NFAT) pathway (22, 23), the protein kinase C (PKC)-IĸB kinase (IKK)-nuclear factor (NF)-κB pathway (24), the Ras-extracellular signal-related kinase (ERK)-activator protein (AP)-1 pathway (25), as well as the Phosphoinositide 3-kinase (PI3K)-AKT-mammalian target of rapamycin (mTOR) pathway (26). These activated signaling pathways ultimately determine cell fate through cytokine production, cell survival, cell proliferation, and differentiation. Although ALK+ ALCLs express rearranged T-cell receptors, the expression of many pivotal TCR molecules, including TCF-1, TCF-1/LEF-1, LCK, ZAP-70, LAT, NFATc1, c-Jun, c-Fos, and Syk is repressed (27). Inhibition of the kinase activity of NPM::ALK or exposure to DNA methyltransferase inhibitors rescues the expression of CD3ε, ZAP70, LAT, and SLP76, suggesting that *NPM::ALK*-mediated transcriptional repression occurs via DNA methylation to downregulate components of the TCR signaling cascade in ALK+ ALCL (28). Apart from this, *NPM::ALK* further mimics TCR-induced signal transduction by directly interacting with and phosphorylating PLC-γ1, which triggers downstream signaling cascades for cell survival (29, 30). As such, NPM::ALK promotes the proliferation and survival of malignant cells by bypassing the TCR signaling pathway (**Figure 1**).

In addition to promoting cell proliferation and differentiation, T-cell receptor engagement also induces activation-induced T-cell death (AICD) through Fas-mediated apoptosis to prevent the accumulation of alloreactive T-cells and the development of graft-vs-host disease (31). It has been demonstrated that FLICE-like inhibitory protein (c-FLIP) prevents neoplastic cells from undergoing Fas-mediated apoptosis in ALK+ ALCL. Specifically, exposure of ALK+ ALCL cell lines that express high levels of c-FLIP, to CH-11, a CD95/FAS agonistic antibody, alone is not able to reduce the viability of malignant cells. si-RNA-mediated knockdown of *c-FLIP* together with CH-11 treatment rescues Fas-mediated apoptosis by triggering downstream caspase signaling pathways in ALK+ ALCL cells (32). Further investigation is required to determine whether the loss of TCR signaling molecules contributes to reduced AICD process in ALK+ ALCL.

### 2.3 Deregulated cell proliferation and apoptosis in ALK+ ALCL

NPM::ALK regulates many proliferative and anti-apoptotic signaling pathways, including mitogen-activated protein (MAP) kinase (33), JAK-STAT (34, 35), PLC-γ1 (30), and PI3K-AKT (29), to promote lymphomagenesis (**Figure 1**). The MAP kinase signaling pathway is a major cell proliferation and survival regulator (36). NPM::ALK phosphorylates extracellular signal-regulated kinase (ERK)1/2 in a MEK1/2-dependent manner, and perturbation of MEK and ERK1/2 reduced cell proliferation and promoted cell apoptosis of ALK+ ALCL cells (37). NPM::ALK also regulates cell growth of ALK+ ALCL via activation of the PLC-γ pathway. The interaction of NPM::ALK with PLC-γ1 occurs via the tyrosine 644 residue, which is located at the C-terminus of the chimeric protein, and expression of NPM::ALK^Y644F^ abrogates PLC-γ1 phosphorylation and activation (30). The PI3K-AKT pathway has been implicated in oncogenesis for its role in cell-cycle progression. NPM::ALK constitutively activates PI3K and its downstream effector, the serine/threonine kinase AKT, and thus promotes growth and inhibits apoptosis in ALK+ALCL (38). AKT phosphorylates the Bcl2-associated death promoter (BAD), thereby suppressing apoptosis and promoting cell survival (39). Similarly, mTOR, a serine/threonine protein kinase and a key regulator of cell growth and proliferation, is also activated by NPM::ALK. Inhibition of the mTOR pathway leads to cell cycle arrest and apoptosis in ALK+ ALCL (40). Further, NPM::ALK promotes the expression of anti-apoptotic factors Bcl-xl and cell cycle-promoting cyclin-dependent kinase 4 (CDK4) and increased levels of phospho-RB to trigger cell proliferation (37). Thus, NPM::ALK regulates cell proliferation and survival while inhibiting apoptosis by orchestrating multiple signaling pathways.

### 2.4 Deregulated cellular metabolism in ALK+ ALCL

Neoplastic metabolic reprogramming is largely characterized by the shift from efficient energy-producing pathways to strategies for biomass production to support cell growth. In this regard, integrated analysis of the phosphoproteomic and metabolomic signature revealed that NPM::ALK signaling triggers an increase in biomass production by rerouting glycolytic intermediates (6) as well as modulation of lipid metabolism, amino acid metabolism, and nucleotide metabolism (**Figure 1**). Particularly, NPM::ALK-mediated phosphorylation of PKM2 at Y105, a key enzyme in aerobic glycolysis, leads to a metabolic switch promoting lymphomagenesis (6). Regarding lipid metabolism, NPM::ALK phosphorylates ATP citrate lyase (ACLY) at residue 682, which may serve as a switch to promote lipid synthesis required for cell proliferation. ACLY is a critical enzyme that catalyzes acetyl-CoA synthesis and connects vital biosynthetic pathways such as carbohydrate and lipid metabolism (41). Genetic or pharmacologic disruption in the NPM::ALK-ACLY signaling axis leads to impaired cell proliferation, impaired clonogenic potential, reduced tumor growth in an *in vivo* xenograft model, and attenuated lipid synthesis in ALK+ ALCL (42). NPM::ALK also modulates cancer metabolism through the downregulation of CD147, causing aberrant glycolysis and thus impairing the major energy source of tumor cells (43).

Further, metabolic alterations in cancer not only modulate the metabolic state of the cell but also impact cellular signaling and the epigenetic state. ALK+ ALCL-derived cell lines and primary tumors exhibit cholesterol auxotrophy due to reduced expression of a critical enzyme, squalene monooxygenase rendering the accumulation of squalene, a metabolite with antioxidant-like properties. Squalene monooxygenase oxidizes squalene to 2,3-oxidosqualene. Aggregation of squalene in cells prevents malignant cells from ferroptosis, which is induced by oxidative stress (44). Cholesterol auxotrophy of ALK+ ALCL can be a therapeutic vulnerability that can be utilized in combination with conventional therapies. In summary, NPM::ALK signaling orchestrates cellular metabolic reprogramming that favors lymphomagenesis.

### 2.5 Increased genomic instability in ALK+ ALCL

Under physiologic conditions, cells consistently encounter intracellular stress, such as reactive oxygen species (ROS) generated by cellular metabolism, and extracellular stress, such as UV light and carcinogenic chemicals. These stresses can disrupt genomic integrity. One of the hallmarks of cancer is genomic instability. It is generally accepted that *NPM::ALK* is required and sufficient to transform primary human T cells in a relatively short span of time. Furthermore, genetic alterations (single nucleotide variants) in ALK+ ALCL are relatively uncommon, suggesting a stable genome (45–47). In ALK+ ALCL, however, impaired DNA repair pathways (48), particularly DNA mismatch repair, may represent a mechanism by which tumor cells initiate additional genetic lesions, considering that a subset of ALK+ALCL patients develop resistance against ALK-specific treatment approaches. To initiate DNA mismatch repair, it is essential for the system to recognize DNA lesions. Two ATPase protein complexes participate in the mismatch recognition process, namely MuTSα, which identifies the base-base mismatches and small insertion/deletion, and MuTsβ, which modulates larger insertion/deletion. MuTSα is composed of MutS protein homolog 2 (MSH2) and MutS protein homolog 6 (MSH6). MuTSβ is composed of MSH2 and MutS protein homolog 3 (MSH3) (49). NPM::ALK directly binds to MSH2 and phosphorylates it at the tyrosine 238 residue. This abnormal interaction prevents MSH2 from binding to its normal partners, MSH3 and MSH6, leading to the ablation of normal MuTs complexes and consequentially impaired DNA damage repair (50). In addition, the expression of *NPM::ALK* in primary CD4+ T cells downregulates genes participating in DNA repair pathways (51). Thus, *NPM::ALK* ablates genomic stability by compromising the DNA mismatch repair process (**Figure 1**). Therefore, the role of *NPM::ALK* in promoting genomic instability by compromising the DNA repair process needs further study.

### 2.6 Epigenetic deregulation in ALK+ ALCL

Epigenetic modifications are heritable yet reversible covalent modifications in DNA or histones that alter the expression of genes without affecting DNA code. The most studied and significant modifications are the methylation of DNA at cytosine residues that function to repress gene expression (52) and the methylation or acetylation of distinct amino acids of the histone tail that dictate their repressive or activating properties (53). These modifications and their combinations dictate nucleosome positioning and local chromatin conformation that provide access to transcriptional regulators to modulate gene expression. DNA modifications direct histone modifications, and methylation of DNA causes steric hindrance to the transcriptional regulators (54). Moreover, the interaction of DNA with methyl-binding proteins, such as methyl CpG binding protein 2 (MeCP2), also prevents transcription factor binding at the locus causing repression of the target gene (52, 55).

*NPM::ALK* regulates the transcriptional silencing of many gene promoters and enhancer regions that encode tumor suppressors through its downstream effector transcription factors (**Figure 1**) (56). NPM::ALK activates the transcription factor STAT3, which upregulates DNA methyltransferase 1 (DNMT1) to methylate target genes for repression (57). As an example, *IL-2Rγ* promoter methylation is induced by *NPM::ALK*. *NPM::ALK* promotes STAT3 binding to the *IL-2Rγ* promoter, which then recruits DNMT1 to its promoter for its silencing (58). Notably, deleting *DNMT1* abrogates lymphomagenesis, suggesting a therapeutic opportunity for targeting DNMT1 in patients who develop resistance to ALK inhibitor treatment (59). ALK+ALCL also exhibits CpG Island methylation at *STAT5A*, a tumor suppressor that reciprocally suppresses *NPM::ALK* gene expression by binding to its enhancer (60). These results show that silencing of tumor suppressor genes by DNA methylation may contribute to the neoplastic transformation of ALK+ ALCL.

In addition to DNA methylation, gene expression is regulated by the chromatin remodeling machinery, which modulates accessibility of the chromatin. Deregulation of chromatin remodelers has been demonstrated to participate in cancer development as well as lymphomagenesis (61, 62). Among them, SWI/SNF is a multi-subunit chromatin remodeling complex that uses the energy generated by ATP hydrolysis to displace or evict nucleosomes and further regulates local chromatin conformation (63). Expression of BRM-Related Gene1 (BRG1), a core component of the human SWI/SNF complex (64), is mediated by NPM::ALK. Further, the expression of BRG1 is dependent on the kinase activity of NPM::ALK. Knockdown of *BRG1* in ALK+ ALCL cells results in a decrease in cell viability compared to scramble shRNA control (65). The role of other chromatin remodelers in the pathogenesis of ALK+ ALCL needs further investigation, and the relationship between the chromatin remodelers and *NPM::ALK* is still largely underexplored.

Loss of cellular identity is intrinsic to neoplastic transformation. ALCLs, despite originating from T-cells, exhibit downregulation of the transcriptional program that defines its T-cell phenotype. The pharmacologic treatment combining DNA demethylation and histone acetylation was insufficient to restore the T-cell phenotype in ALK+ ALCL cells. This suggests that additional stimulus is required to repress the T-cell phenotype. However, other T-cell lymphoma cells exposed to the same treatment exhibited expression of genes characteristic of ALCL (*ID2*, *LGALS4*, *c-JUN*) as well as loss of T-cell phenotype marked by loss of *CD3*, *LCK*, and *ZAP70* expression indicating that global DNA demethylation and histone acetylation are critical for cellular reprogramming towards an ALCL-like phenotype (66).

The combinatorial pattern of DNA methylation and histone post-translational modifications (PTMs) are increasingly appreciated as epigenetic signatures of cancer subtypes. These modifications regulate cellular processes, such as cell cycle regulation, apoptosis, and DNA damage response (67–70). *BCL2L11*, also known as *BIM* (Bcl-2 interacting mediator of cell death), a Bcl-2 homology 3 (BH3)-only proapoptotic protein that belongs to the Bcl-2 family, is epigenetically silenced via the combinatorial deregulation of DNA methylation and histone acetylation in ALK+ ALCL (66, 71). Recruitments of MeCP2 and Sin3a/histone deacetylase1/2 (HDAC1/2) corepressor complex to the *BIM* promoter contributes to its silencing. Exposure of the DNA methylase inhibitor, 5-azacytidine, or the HDAC inhibitor, trichostatin, alone to ALK+ ALCL cells is not only able to rescue the expression of BIM at both mRNA and protein level but also increases apoptosis (71). This suggests that DNA methylation and histone acetylation together may contribute to the pathogenesis of ALK+ ALCL. In this regard, the pharmacological modulation of altered epigenetic machinery may represent novel therapeutic interventions.

Histone PTMs alone can also dictate disease-specific changes in the transcriptional program, and the pattern of histone PTMs can be utilized as a novel biomarker of disease subtypes (72). HDAC inhibitors have already been approved by the Food and Drug Administration (FDA) for the treatment of T-cell malignancies (73–75). However, the comprehensive landscape of histone PTMs, such as methylation and acetylation (66, 76), phosphorylation (77), ubiquitination (78), and sumoylation (79) for different classes of ALCL is yet to be determined. Since the current therapeutic approach of using HDAC inhibitors has been shown to cause nonselective toxicity, further understanding of the comprehensive epigenetic landscape of ALCL is warranted as it may lead to discoveries of novel histone modifications and their writers and erasers, which can be targeted for precision therapeutics (80). Evaluation of ALCL subtypes with highly sensitive proteomic approaches for histone modification analysis as well as single-cell proteomic approaches with enrichment and analysis of histone PTMs will add significant value to define the epigenetic signature of the disease.

### 2.7 Deregulated MicroRNA repertoire in ALK+ ALCL

It has been observed that nearly 90% of the human genome is transcriptionally active, yet only 1.4% of this transcriptome is constituted by protein-coding mRNA (81). The role of non-coding RNAs (ncRNA) is underappreciated yet critical to cell physiology and diseases, including ALCL. In ALK+ ALCL, the fusion protein NPM::ALK is associated with non-coding RNAs (ncRNAs), such as microRNAs to alter the gene expression signature of ALK+ ALCL (**Figure 1**) (82, 83). Along with tRNA and ribosomal RNA, the non-coding transcriptome is comprised of small nuclear RNA (snRNA), long noncoding RNA (lncRNA), and microRNA (miRNA). MicroRNAs are short, usually 20-23 nt long non-coding RNA that function by activating the RNA-induced silencing complex (RISC) against specific mRNA targets (84). miRNA array based on locked nucleic acid (LNA) technology containing 636 human and 425 murine miRNA probes performed on ALK+ ALCL cell lines identified distinct miRNA clusters from ALK+, to ALK- ALCL. These clusters are cross-validated with *Npm::alk* transgenic mice and primary ALK+ and ALK- ALCL to classify the miRNA unique to each disease group. These studies demonstrated strong upregulation of the miR-17-92 cluster in ALK+ ALCL and miR-155 upregulation in ALK- ALCL. Further, reduced expression of miR-101 is observed in both ALK+ ALCL and ALK- ALCL (82). Subsequent studies identified 32 miRNAs associated with ALK expression *in vitro*, presenting distinct miRNA expression profiles (85). These studies identify 7 miRNAs, of which 5 are upregulated (miR-512-3p, miR-886-5p, miR-886-3p, miR-708, miR-135b) and 2 downregulated (miR-146a, miR-155) in ALK+ ALCL. Another similar study identifies a distinct profile of miRNA that are specific to ALK+ or ALK- ALCL and cross-validated earlier findings. Moreover, it also identifies that miR-181a, which participates in the regulation of T-cell differentiation and TCR signaling, is significantly downregulated in ALK+ ALCL (86).

The role of exosomal miRNA in promoting disease dissemination of ALK+ ALCL has been recently reported. RNA sequencing studies identified 12 miRNAs that are significantly differentially expressed in the plasma of 20 NPM::ALK+ ALCL patients compared to healthy donors (n=5). Among these miRNAs, the level of miR-122-5p has further been validated as highly expressed in a larger cohort of ALCL patients (n=66) compared with healthy donors. Levels of miR-122-5p are elevated in late-stage (III-IV) ALCL patients compared to those with early-stage (I-II) disease. Interestingly, the expression of miR-122-5p is barely detectable in lymph nodes and other tissues but highly enriched in the liver of ALCL patients. *In vitro* and *in vivo* experiments indicate that miR-122-5p expressed in small extracellular vesicles promotes the proliferation and progression of ALCL cells (87). These mechanisms employed by miRNA using small extracellular vesicles for the pathogenesis of ALK+ ALCL may represent opportunities for discovery of novel mechanisms of disease dissemination as well as identification of prognostic biomarkers.

### 2.8 Immune evasion in ALK+ ALCL

Immune evasion by cancer cells is increasingly appreciated as an emerging hallmark of cancer. ALK+ ALCL cells exploit molecular mechanisms that bypass immune recognition (**Figure 1**). NPM::ALK-STAT3 signaling in ALK+ ALCL induces expression of transforming growth factor beta (TGF-β), IL-10, and cell surface receptor PD-L1 (CD274, B7H1), creating an immunosuppressive tumor microenvironment (88). The NPM::ALK-STAT3-DNMT1 pathway also epigenetically downregulates CD48, an immune surveillance molecule, to prevent tumor cell recognition by natural killer cells. STAT3 directly binds and methylates the promoter of *CD48* in association with DNMT1. Pharmacologic inhibition of NPM::ALK, STAT3, or DNMT1 sensitizes ALK+ ALCL towards NK cell-mediated cytotoxicity *in vitro*. Further, expression of CD48 in ALK+ ALCL cell line increases NK cell-mediated cytotoxicity *in vitro* and in a xenograft mouse model (89). Similarly, NPM::ALK-STAT3 pathway induces the expression of *ICOS*, a member of the CD28 costimulatory receptor superfamily, by transcriptional induction, as well as suppresses the ICOS inhibitor miR-219 (90). Since ICOS engagement promotes ALK+ ALCL proliferation, it is tempting to speculate that by engaging its ligand (ICOS-L), tumor-specific ICOS subverts other critical co-stimulatory signals from immune cells, impairing cytotoxic response to tumor cells. Previous studies suggest that ALK+ ALCLs and ALK+ ALCL cell lines, do not express TNF-α as a result of promoter methylation, thus preventing its proapoptotic function on tumor cells (91). Importantly, inhibition of DNMT1 by 5’-aza-2’-deoxy-cytidine (5-ADC) rescues the expression of *TNF-α* mRNA and protein. Further, exogenous TNF-α expression inhibits the growth of ALK+ ALCL cell lines and induces the activation of apoptotic pathway intermediates, namely caspase 8 and caspase 3. Hence, inhibition of DNMT1 not only triggers the NK cell-mediated cytotoxicity but also promotes the proapoptotic signaling pathway in ALK+ ALCL, raising the possibility of DNA methyltransferase inhibitors as a therapeutic option for ALK+ ALCL. The observation that the serum titers of anti-ALK antibodies in patients are inversely proportional to stage stratification and progression of disease indicates that NPM::ALK protein is immunogenic and triggers a natural immune response that keeps a check on disease progression to some extent (92). Therefore, it will be important to comprehensively investigate NPM::ALK-mediated immune escape mechanisms. A better understanding of the immune evasion mechanism will help in developing potential alternative or combinatorial therapeutic interventions for ALK+ ALCL.

### 2.9 Deregulation of transcription factors in ALK+ ALCL

Various models have been proposed for the origin of malignant cells in ALK+ ALCL. The expression of CD4 or CD8 and CD30, along with clonal T-cell receptor (TCR) rearrangement, suggests that the malignant cells may originate from activated T cells (93), while the expression of FoxP3, IL10, and TGFβ suggests a regulatory T cell origin (88), and BATF and BATF3 expression is associated with a Th17/group 3 innate lymphoid cell origin (94). In addition, *NPM::ALK*-transformed CD4+ T lymphocytes and primary ALK+ ALCL biopsies share characteristics with early T cell precursors (51). Further, ALK+ ALCL cells overexpress stem cell transcription factors (*OCT4*, *SOX2*, and *NANOG*) and *HIF2A*, which regulate hematopoietic precursor differentiation and cell growth. These findings suggest that NPM::ALK signaling may trigger dedifferentiation to early thymic progenitor-like characteristics in CD30+ mature CD4+ T cells (95). In another study utilizing the RAG2^-/-^ mice model, which lacks the machinery to produce mature T or B cells (96), it was shown that *NPM::ALK* is capable of promoting thymic T cell maturation and TCR-independent tumor formation, suggesting that the initial stage of ALK+ ALCL development may occur in the thymus (97).

Further, constitutive activation of STAT3 is highly prevalent in ALK+ ALCL and contributes to its pathogenesis. *NPM::ALK* interacts and phosphorylates STAT3 leading to its activation and nuclear translocation, where it regulates the transcription of a number of genes known to be involved in apoptosis and cell cycle progression (**Figure 1**) (35, 98). In ALK+ ALCL, the activation of STAT3 is multifactorial. JAK3, a major physiologic activator of STAT3, is highly activated in ALK+ ALCL lines and primary tumors (34). JAK3 interacts with NPM::ALK, and its inhibition decreases the tyrosine kinase activity of NPM::ALK (99, 100). Constitutive activation of STAT3 in ALK+ ALCL is also contributed by the downregulation of SH2 domain-containing protein tyrosine phosphatase-1 (SHP1) in ALK+ ALCL (101, 102). SHP1 interacts with JAK and NPM::ALK and dephosphorylate crucial tyrosine sites and thus inhibits the kinase activity (101, 103). ALK+ ALCL from children and adult patients exhibit loss of *SHP1* at a frequency of 50% and 86%, respectively. Further, *SHP1* is methylated and thus silenced in a number of ALK+ ALCL cases (101, 102).

ALK+ ALCL cells also aberrantly express multiple members of the activator protein-1 (AP-1) family of transcription factors, which includes proteins of the Jun, Fos, ATF, and Mf subfamilies (104). AP-1 family proteins regulate a wide range of cellular and biological activities, including cell cycle and proliferation, apoptosis, autophagy, and lipid synthesis (105). They also regulate cell migration and invasion as well as inflammatory response and immune cell development and activation. Studies have shown that AP-1 proteins play a pivotal role in promoting cell survival, proliferation, and suppression of AP-1 proteins can lead to apoptosis in ALK+ ALCL (94, 106, 107). Since AP-1 family proteins regulate a myriad of signaling pathways, further investigation will be required to comprehensively understand their impact on the ALK+ ALCL pathogenesis.

In addition, C/EBPβ, CCAAT enhancer binding protein, a transcription factor that belongs to the C/ECP leucine zipper transcription factor family, is highly expressed in ALK+ ALCLs (108). The overexpression of C/EBPβ is mediated through the NPM::ALK-STAT3 axis and is dependent on the kinase activity of NPM::ALK (108, 109). Moreover, NPM::ALK also fosters stability and translation of *C/EBPβ* mRNA via enhancing binding of AU-binding protein HuR to the 3′-UTR of *C/EBPβ* transcript (110). C/EBPβ modulates gene expression and miRNA levels to promote the transformation, proliferation, and survival of the malignant cells in ALK+ ALCL (111, 112). Therefore, targeting the deregulated transcription factors and the signaling pathways regulated by them may serve as novel therapeutic interventions for ALK+ ALCL.

### 2.10 Deregulated cytokine signaling in ALK+ ALCL

Cytokine and cytokine receptor signaling orchestrate the immune response, hematopoiesis, cell differentiation, and cell growth (113). There is an aberrant cytokine repertoire in ALK+ ALCL (**Figure 1**). Integrated unbiased N-glycoproteomic and transcriptomic profiling of 32 different B cell, T cell, and NK cell lymphoma cell lines has identified many cytokine receptors, including the interleukin receptor IL-R, as well as T helper (Th) receptors, expressed by ALK+ ALCL cells (8). Similarly, the level of IL-2R, Oncostatin M (OSM), IL-6, IL-8, IL-9, IL-10, IL-17a, IL-22, and soluble CD30 is decreased in either pediatric or adult ALK+ ALCL patient samples after they reached complete remission (114–116). There is a correlation between stages of the disease, presence of the minimal disseminated disease, anti-ALK antibody titers, and risk of relapse with concentrations of cytokines including IL-6, interferon-γ (IFN-γ), IFN-γ induced protein as well as sIL-2R among ALK+ ALCL pediatric patients (114). Moreover, levels of IL-6 demonstrated an independent prognostic value with a hazard ratio of 2.9 ± 0.4.

In addition, exogenous *NPM::ALK* expression leads to significant reductions of GM-CSF, TNF, and IL2 (51). Inhibition of NPM::ALK reduces the expression of cytokine receptor proteins, including IL-1R1, IL-1R2, IL-1RAP, IL-2RA, IL-4, IL-18RA, and IL-31RB (8). These observations suggest that constitutively activated ALK signaling contributes to deregulation of cytokine signaling.

Functional studies reveal that *NPM::ALK* regulates multiple JAK-STAT pathways, including IL-2/STAT5, and IL-6/STAT3 to participate in the aberrant cytokine secretion in ALK+ ALCL (8, 117). Particularly, *NPM::ALK* induces upregulation of *STAT3* and *STAT5* expression, which upregulates IL-31RB in ALK+ ALCL (118). In addition to STATs, *NPM::ALK* also enhances cytokine production by inducing the expression of other transcription factors, such as AP-1. AP-1 binds to promotors of multiple cytokines and thus regulates *IL17F, IL22, IL26, and IL23R* genes in ALK+ ALCL (94, 119).

Besides activation, *NPM::ALK* also deregulates the cytokine signaling pathway by suppressing transcription factor function. Among normal human endothelial cells, STAT1 is one of the major modulators of IFN-γ, which can further antagonize IL-6-mediated STAT3 activation (120). During activation, STAT1 forms a homodimer. It can also bind with *STAT3* and form a heterodimer. The gene expression levels and specificities are modulated by the STAT1 homodimer vs heterodimer ratio (121). In ALK+ ALCL, *NPM::ALK* also downregulates STAT1 to antagonize STAT3 and further decrease the production of antitumor cytokine IFN-γ (122).

Further, epigenetic modulation also contributes to cytokine deregulation in ALK+ ALCL. It has been reported that *NPM::ALK* downregulates SHP1 tyrosine phosphatase, a negative modulator of multiple cytokine signaling pathways, including Epo-R, IL-4, IL-13, IL-3R, IL-2R, through STAT3-mediated upregulation of DNA methyltransferase 1 in ALK+ ALCL (102, 123, 124).

The tumor microenvironment also contributes to the formation of deregulated cytokine repertoire (125, 126). However, the composition and cross-talk between the neoplastic cells and tumor microenvironment of ALK+ ALCL need further investigation.

### 2.11 Deregulated proteolysis in ALK+ ALCL

Deregulated proteolysis by ubiquitination or sumoylation contributes significantly to the sustained signaling of oncogenic proteins (127, 128). The proteasomal degradation process of target proteins requires small ubiquitin binding to the substrate (127). Similarly, SUMOylation is another post-translational modification characterized by the reversible conjugation of small ubiquitin-like modifiers (SUMOs) with the target protein. SUMOylation modification often competes with ubiquitin for substrate binding and is believed to protect candidate proteins from proteasomal degradation (128). Studies suggest that the SUMOylating of NPM::ALK antagonizes its ubiquitination and subsequent degradation prolonging its oncogenic signaling (129). Further, the removal of sumoylation by SENP1 (a sentrin-specific family of proteases) promotes NPM::ALK protein turnover and ensues a decrease in cell viability, cell proliferation, and colony formation ability. It can be surmised that targeting NPM::ALK degradation may have therapeutic benefits in ALK+ ALCL that are resistant to NPM::ALK kinase inhibitors. In this regard, several efforts are underway to develop ALK protein degraders at different levels of preclinical or clinical settings (130–133).

## 3 Pathobiology of ALK- ALCL

ALK- ALCL is a CD30+ large T-cell lymphoma that typically affects the older population and has variable prognosis (134, 135). Currently, ALK- ALCL is subdivided into three classes, namely systemic ALCL, breast implant-associated ALCL, and primary cutaneous ALCL. Depending upon the genetic lesions acquired, the pathogenic mechanisms and disease aggressiveness may vary.

### 3.1 Pathobiology of systemic ALK- ALCL

**Figure 2:**
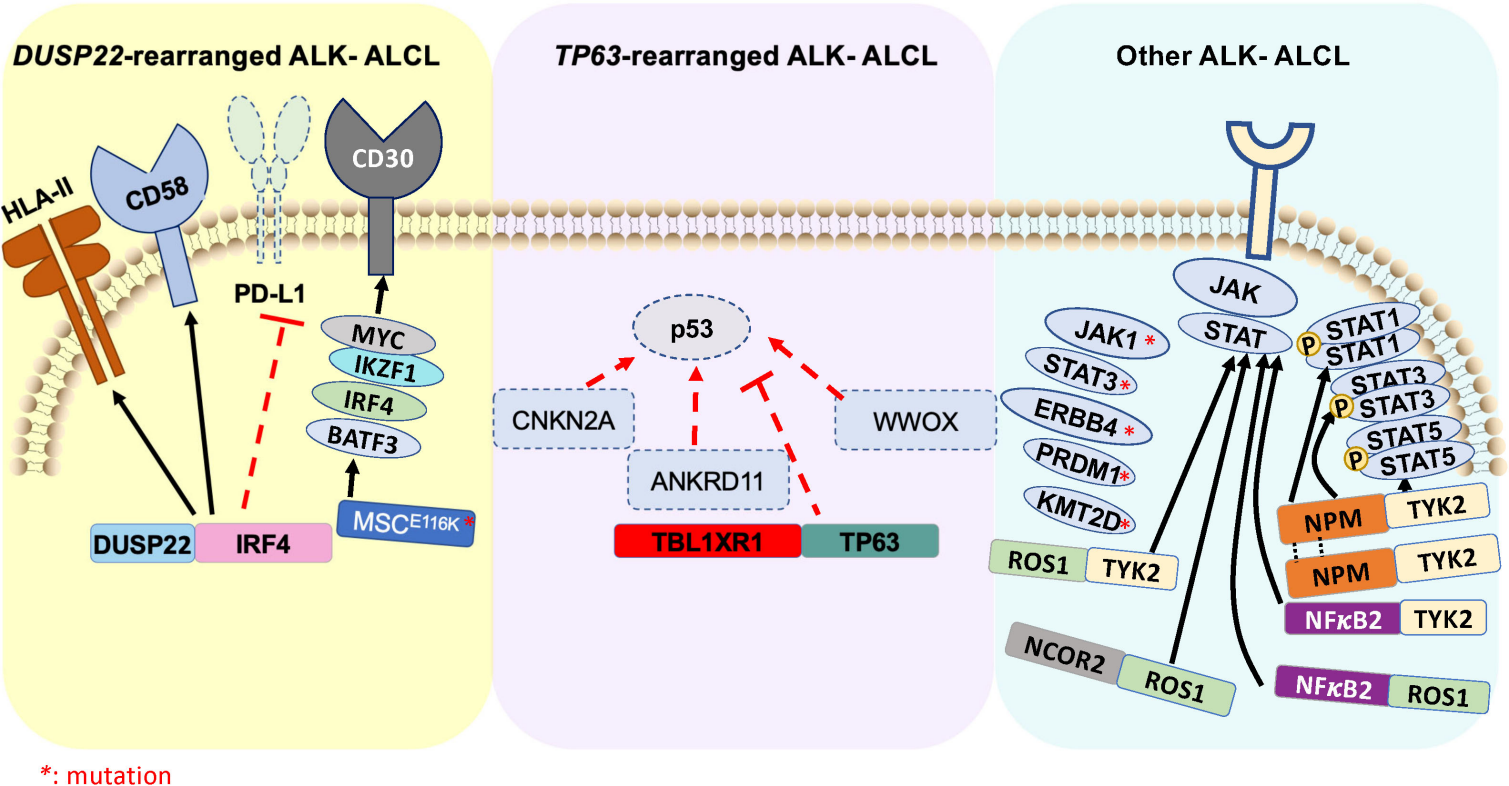
Schematic illustration of genomic rearrangements that contribute to pathogenesis of ALK- ALCL (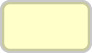) represents ALK- ALCL harboring *DUSP22*-rearrangement and associated signaling pathways. (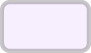) represents ALK- ALCL harboring *TP63*-rearrangement and associated signaling pathways. (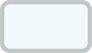) represents various driver oncogenic fusions involving tyrosine kinase and transcription factors, as well as high-frequency mutations that constitute an independent subgroup of ALK- ALCL.

In ALK- ALCL, two gene rearrangements and identified recurrent mutations subclassify ALK- ALCL into three more categories, namely fusion involving *DUSP22::IRF4*, fusions involving *TP63* gene, and other types of ALK- ALCL (**Figure 2**).

#### 3.1.1 *DUSP22*-rearranged ALK- ALCL

Rearrangement of *DUSP22* occurs near the *DUSP22::IRF4* locus on 6p25.3 (136). The FRA7H fragile site on 7q32.3 is the most affected gene in the translocation t (6;7)(p25.3,q32.3). *DUSP22* rearrangements are detected at a frequency of 30% in ALK- ALCL cases using fluorescence *in situ* hybridization (FISH). This rearrangement leads to downregulation of DUSP22, and upregulation of the microRNA miR-29 on 7q32.3 but does not affect the expression of IRF4. This subgroup lacks the expression of genes associated with JAK-STAT3 signaling, but results in overexpression of the immunogenic cancer-testis antigen, marked DNA hypomethylation, and exhibits a reduced expression of PD-L1 and high expression of CD58 and HLA class II (137). Further, a novel recurrent mutation in *MSC* ^E116K^, a gene encoding musculin, has been recently identified in *DUSP22* rearranged ALK- ALCL. This mutation induces the CD30-IRF4-MYC signaling axis (**Figure 2**) and drives cell proliferation (138). Interestingly, DUSP22 inhibits interleukin-6 (IL-6)-induced STAT3 activation, and its downregulation is another mechanism by which STAT3 signaling may be activated in ALK- ALCL (139). Notably, the 5-year overall survival of *DUSP22*-rearranged cases is approximately 85-90%, which is significantly higher than other ALK- ALCL (140).

#### 3.1.2 *TP63*-rearranged ALK- ALCL

The *TP63* gene, a member of the p53 family, is expressed either as a full-length isoform carrying a transactivator domain (TP63) or as an amino-deleted isoform (ΔNp63) (141). P63 triggers multiple signaling in cancer-specific contexts, including regulation of the cell cycle, apoptosis, stemness, and tumorigenesis (142). Approximal 8% of ALK- ALCL show rearrangement of *TP63* in 3q28, frequently with *TBL1XR1* as a result of an inversion (3)(q26q28) (**Figure 2**) (143). Rearrangements involving *DUSP22* and *TP63* are mutually exclusive. *TP63*-rearranged ALK- ALCL exhibits the worst prognosis within the ALCL subtypes, with a 5-year overall survival rate of 17%. The biological significance of the genetic rearrangement of *TP63* in ALK- ALCL is yet to be determined.

#### 3.1.3 Other types of ALK- ALCL

In ALK- ALCL, oncogenic mutations in *JAK1* and/or *STAT3* (**Figure 2**), which contribute to the consistent activation of the STAT3 signaling pathway, has been identified in nearly 20% of cases. In addition, oncogenic fusion genes involving a transcription factor and a tyrosine kinase, such as *NFκB2::ROS1*, *NCOR2::ROS1*, *NFκB2::TYK2*, and *PABPC4::TYK2* have been identified in ALK- ALCL (144). These fusion chimeras result in increased STAT3 activity and develop ALCL phenotype via STAT3 signaling, suggesting that intercepting STAT3 activation may have a therapeutic advantage (94). A recent deep-targeted next-generation sequencing of 47 ALK+ and 35 ALK- ALCL demonstrated that, on average, ALK- ALCL harbor 4.2 mutations/patient compared to 2.6 mutations/patient for ALK+ ALCL. Among all the mutations, *STAT3* and *JAK1* mutations are the most frequent (26%) in ALK- ALCL. The mutations that predicted poor prognosis of ALK- ALCL includes *TP53, STAT3, EPHA5, JAK1, PRDM1, LRP1B, and KMT2D* (46).

Approximately 25% of ALK- ALCL expresses an oncogenic truncated ERB-B2 receptor tyrosine kinase-4 (ERBB4) that is not detected in ALK+ ALCL and PTCL-NOS and may likely form another subgroup of ALK- ALCL. ERBB4 expression is mutually exclusive of *DUSP22*, *TP63*, and *ROS1* rearrangements. Pharmacologic inhibition of ERBB4 partly controls ALCL cell growth and disease progression in an ERBB4-positive patient-derived tumor graft model (145).

Hence, better understanding and targeting these rearrangements and mutation-mediated signaling pathways may serve as novel therapeutic interventions for different subtypes of ALK- ALCL.

### 3.2 Pathobiology of breast implant-associated ALCL

Breast implant-associated ALCL (BI-ALCL) is a distinct subtype of mature T-cell lymphoma. A persistent chronic inflammation occurring post-breast implants, particularly those with a textured outer shell, has been documented as the underlying cause of the disease (146). Cross-talk between the malignant cells and reactive cells in the microenvironment is thought to contribute to the formation of an inflammatory milieu characteristic of BI-ALCL. Elevated levels of IL-1β, IL-6, and TNF-α, the macrophage-activating cytokines, have been detected after culturing peripheral blood mononuclear cells obtained from healthy donors to the surfaces of the silicone breast implants for 4 days (147). However, no T-cell activation or specific effector cell subtype skewing has been observed. In addition, elevated expression of IL-13, IgE+ eosinophils, and mast cells in the microenvironment of primary BI-ALCL specimens suggests that allergic inflammation may contribute to the development of BI-ALCL (148).

Tumors display complex karyotypes with losses of chromosomes 1p,4q, 8p, 10p, 15, 16, 20 and gain of chromosomes 2, 9p, 12p, 19p, and 21 in BI-ALCL patients (149, 150). Targeted sequencing of 180 genes in 11 cases identified highly recurrent activating *STAT3* mutations and recurrent deletions of 1p22 involving *RPL5*, a tumor suppressor that regulates cell proliferation. In addition, abnormalities were identified in TGF-β, PKC, WNT/β-catenin pathway, and inflammasome signaling. Amplifications involving *TNFRSF11A* and *PDGFRA* were also identified (151). Genomic profiling of BI-ALCL using a variety of sequencing platforms did not detect any genomic rearrangements involving *ALK*, *DUSP22*, and *TP63*, suggesting less heterogeneity in the genetic manifestation than other subtypes of ALCL (152). Predominant JAK-STAT pathway, TP53, and DNMT3A could be molecular drivers of BI-ALCL (153). JAK1 mutations were found in 13% (3/23) of cases, with the most frequent point mutation involving G1097(D, V or S) identified in 44% (4/9) of cases (152, 154, 155). The frequency of *STAT3* mutations was 26% (6/23), with the most predominant mutations identified involving S614R (155). Apart from the JAK-STAT pathway, the second most frequent alterations in BI-ALCL were identified in epigenetic modifiers, including *TET2, TET3, ARID4B, KDM5C, KDM6A, KMT2C/D, CHD2, CREBBP* and *SMARCB* at the frequency of ~55-75% (150, 154). Currently, the first line of therapy involves surgical removal of the implant in combination with radiotherapy and standard chemotherapy. However, therapeutic targeting of JAK/STAT pathway and epigenetic deregulations may be considered as alternative therapeutic opportunities for BI-ALCL.

## 4 Pathobiology of primary cutaneous anaplastic large cell lymphoma

Primary cutaneous anaplastic large cell lymphoma (pc-ALCL) is a CD30+ lymphoproliferative disorder that manifests in the skin. The malignant cells exhibit large anaplastic and pleomorphic morphology with expression of CD30 in approximately 75% of cells. It has a relatively good prognosis in the absence of high-stage disease. The disease is currently classified as a subtype of primary cutaneous lymphoid proliferations and lymphomas that encompass a spectrum of other diseases, including lymphomatoid papulosis (LyP) (5). Morphologic features of pc-ALCL partly overlap with other diseases, such as LyP (156–158) and reactive lymphoid hyperplasia (159). Therefore, genetic characterization of the disease is critical for correct diagnosis.

The majority of pc-ALCLs lack genomic rearrangements in *ALK, DUSP22*, and *TP63*. Although unusual, ALK-positive cases with only skin lesions have been identified, the frequency of these cases and ALK fusion partners are yet to be further determined (156, 160, 161). Array comparative genomic hybridization analysis of pc-ALCL demonstrates that nearly 40% of cases exhibit chromatin imbalances targeting region encompassing genes *RAF1* (3p25), *CTSB* (8p22), *FES* (15q26.1), *FGFR1* (8p11), *NRAS* (1p13.2), *MYCN* (2p24.1), and *CBFA2* (21q22.3) (162). Further, highly recurrent genomic loss of chromosomes 6q16-6q21, 6q27, and 13q34, as well as gain on the chromosome 7q31 and 17q, were also detected (163). In addition, a recurrent translocation involving *IRF4::MUM1* at chromosome 6p25.3 was identified at the frequency of approximately 20 to 25% in pc-ALCL. However, the protein expression of IRF4 and MUM1 is also detected in systemic ALCL, and therefore, examining the expression of IRF4 and MUM1 by IHC does not reliably distinguish pc-ALCL from systemic ALCL (157). Further, we identified a novel recurrent *NPM::TYK2* gene fusion in a proportion of primary cutaneous CD30+ lymphoproliferative disorders (15%), which activates STAT1/3/5 signaling and promotes cell proliferation (164). Importantly, a transgenic conditional knock-in Cd4-CreNPM::TYK2^fl/fl^ mouse model demonstrates spontaneous development of CD30+ mature T-cell lymphoma with 90% penetrance (165). Hence, targeting TYK2 may serve as a therapeutic intervention for neoplasms harboring the *NPM::TYK2* rearrangement.

## 5 Conclusions and future perspectives

Our understanding of ALK+ ALCLs has provided opportunities for targeted therapies such as small molecular inhibitors of ALK (crizotinib, alectinib, and ceritinib) and antibody-drug conjugates targeting the tumor-specific expression of CD30. Given that a significant fraction of patients experience relapse or refractory responses, there is a continued need for the development of novel therapeutic approaches that target aberrant signaling and/or immune evasion mechanisms. ALK- ALCLs remain a genetically heterogeneous group of mature T-cell lymphoma. The identification of gene rearrangements involving *TYK2*, *DUSP22*, *TP63*, and *ERBB4*, and genetic alterations characteristic of distinct subsets of ALK- ALCLs, will facilitate improved stratification of disease outcomes. The discovery of novel gene rearrangements within the ALK- ALCL category and their functional consequences will be crucial for precision therapeutics. BI-ALCL demonstrates a predominant role of activated JAK-STAT3 signaling as the major driver of disease partly due to recurrent point mutations in *JAK1* and *STAT3*. Further, the contribution of epigenetic modifiers in conjunction with JAK-STAT3 signaling in the propagation of BI-ALCL has not been functionally explored and warrants further investigation. Moreover, an integrated approach of genetic, epigenetic, and proteomic profiling may offer an opportunity to identify novel therapeutic targets for ALCL. Despite studies that have identified the role of cytokine deregulation in ALCL, the composition of the microenvironment and its role in regulating tumor cell survival mechanisms remains largely unexplored.

## Author contributions

RW and ML contributed to the ideas and structure of the manuscript. RW and ML wrote the review. All authors contributed to the article and approved the submitted version.
